# Failure of XEN Gel Stent Implantation as a Stand-Alone Procedure in Congenital Glaucoma: Case Report of Secondary Congenital Glaucoma in Neurofibromatosis Type 1

**DOI:** 10.1155/2021/9947167

**Published:** 2021-07-23

**Authors:** Hannah Schellhase, Matthias Fuest, David Kuerten, Peter Walter, Niklas Plange

**Affiliations:** Department of Ophthalmology, Universitätsklinikum Aachen, Pauwelsstr. 30, 52057 Aachen, Germany

## Abstract

A XEN gel stent implant procedure was performed in a one-year-old child with severe unilateral congenital glaucoma. At the age of 6 weeks, an uncomplicated 360° trabeculotomy had been performed, which resulted in intraocular pressure (IOP) control for only 4 months. The gel stent implantation was performed ab interno without complications. However, 1 month later, the stent was repelled into the anterior chamber due to the elasticity of Tenon's layer. A first revision surgery was performed, with excision of Tenon's layer and implantation of a new gel stent under sight. At the age of 18 months, a second revision surgery was performed because of an encapsulated Tenon cyst with insufficient IOP control, again with the implantation of a new stent. At that time, a progressive upper eyelid swelling was apparent. Eyelid biopsy led to the diagnosis of neurofibromatosis type 1, presenting with an orbital plexiform neurofibroma. Further insufficient IOP control resulted in a cyclodestructive procedure and loss of light perception during follow-up. XEN gel stent implantation in congenital glaucoma in infants is more challenging than that in adult patients. Gel stent implantation ab interno may be difficult due to the thickness and elasticity of Tenon's layer. Gel stent dislocation may occur, even months after surgery. Trabeculectomy might be a better approach after failed trabeculotomy in congenital glaucoma. An underlying systemic disease might become apparent late during follow-up.

## 1. Introduction

Congenital glaucoma is a rare disease but still an important cause of childhood ocular morbidities. Typical clinical signs are corneal clouding, megalocornea, epiphora, and buphthalmos. Paediatric glaucoma surgery is the first treatment of choice. However, it is challenging due to the differences in anatomy and tissue behaviour, compared with adult eyes. Angle surgery, such as trabeculotomy, is the preferred initial surgical option for congenital glaucoma; it has high success rates and a good safety profile [1, 2]. However, in case of failure of the initial intervention, further surgical treatment becomes more challenging. Then, trabeculectomy and tube shunt implantation may be necessary, with challenging bleb-related postoperative care. Ciliodestructive procedures are considered to be options of last resort [3, 4].

The XEN gel stent (Allergan, Dublin, Ireland) is a tubular implant designed to affect IOP control with a filtering bleb, as an alternative to conventional trabeculectomy. The implantation is performed ab interno using a minimally invasive approach, avoiding surgical manipulation of the conjunctiva. However, bleb encapsulation often occurs and bleb revision may be necessary in 28% of pseudophakic and up to 50% of phakic adult patients [5].

We report the failure of XEN gel stent implantation in a child with unilateral secondary congenital glaucoma in neurofibromatosis type 1 (NF1). We discuss the implications for possible use of the XEN gel stent in children.

## 2. Case Presentation

**Figure 1:**
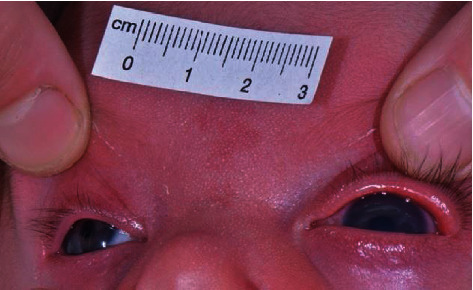
Photograph of the patient at age 6 weeks. The left eye shows buphthalmos and corneal oedema.

A 6-week-old female was referred for glaucoma evaluation after the mother had identified left eye enlargement and tearing. The newborn's medical history was uneventful, with full-term delivery. No family history of congenital glaucoma or other inherited diseases was evident. On examination, the child was unable to fixate with the left eye (OS) and strongly resisted occlusion of the right eye (OD). A buphthalmos OS was apparent, with a large corneal diameter and cloudy corneal stroma (Figure 1). Examination under anaesthesia (EUA) was performed, revealing suspected unilateral congenital glaucoma OS. After the induction of anaesthesia, IOP was measured as 11 mmHg OD and 40 mmHg OS using iCare rebound tonometry (iCare® PRO, Icare, Vanda, Finland). All IOP measurements during further examinations were performed using this rebound tonometer. Corneal diameters were 10 mm horizontally OD and 12 mm horizontally OS. Handheld slit lamp examination revealed a clear cornea OD and corneal oedema and extensive Haab's striae OS. Corneal thickness, measured using ultrasound pachymetry, was 550 *μ*m OD; OS could not be measured due to extensive corneal oedema. The iris did not show any structural abnormalities, and no ectropium uveae was apparent. Gonioscopic evaluation of the OS was not possible due to the corneal oedema. Dilated fundus examination showed a healthy optic nerve with a cup-to-disc ratio of 0.4 OD and, due to limited visibility, a presumed terminal excavation OS. Axial lengths, measured using A-scan ultrasound, were 16.5 mm OD and 20.5 mm OS.

The patient was diagnosed with congenital unilateral glaucoma and buphthalmos OS. She underwent 360° trabeculotomy using an illuminated microcatheter (iTrack™250, Ellex, Minneapolis, MN, USA). A paediatric examination at 6 weeks of age did not reveal any systemic disease or other ocular or nonocular malformations. Over the next 4 months, IOP ranged from 14 to 20 mmHg OS. The corneal oedema declined significantly. Amblyopia treatment with occlusion was not tolerated because of severe amblyopia and advanced glaucomatous optic disc damage.

Five months posttrabeculotomy, IOP was found to be elevated in OS up to 30 mmHg. Travoprost and dorzolamide-timolol in fixed combination twice daily were prescribed to lower IOP. The patient underwent a second EUA. The angle OS was closed, with anterior synechia on gonioscopy. We assumed that anterior synechiae had developed after the 360° trabeculotomy, although no gonioscopic evaluation was possible at the initial examination due to the corneal oedema. Therefore, a 360° synechiolysis of the anterior synechia was performed using a standard surgical spatula with a gonioscopic lens with two paracenteses. This intervention produced satisfying IOP (up to 21 mmHg) for 3 months. This surgical intervention had to be repeated 3 months later, after IOP had again increased and angle OS was closed on gonioscopy.

After another rise in IOP (39 mmHg OS at age 1 year), we decided to perform a filtering surgery using the XEN gel stent via a conventional ab interno approach, along with subconjunctival injection of 0.1 mL mitomycin C (MMC) 0.2 mg/mL. We assumed that XEN implantation might incur less surgical trauma than a standard trabeculectomy, especially considering postsurgical complications. The XEN gel stent procedure is a minimally invasive glaucoma surgery that affects a defined externalisation of the aqueous humour to a subconjunctival filtering zone. The XEN gel stent is designed as an advancement of the trabeculectomy, with less risk of severe complications. Over the next month, IOP ranged from 8 to 15 mmHg OS without use of medication, and the patient showed a diffuse functional filtering bleb. No adverse events were present, such as flattening of the anterior chamber or choroidal detachment.

However, 1 month later, increased IOP was again apparent. EUA revealed the XEN implant to be dislocated too far into the anterior chamber. About 50% of the XEN implant was visible in the anterior chamber; the implant was still in contact with the inner part of the scleral tunnel. During this first XEN revision surgery, the implant could not be repositioned without opening the conjunctiva because of the strong Tenon's layer. The stent was removed, and a new XEN gel stent was implanted under sight ab interno after tenonectomy had been performed. The new XEN implant was positioned in the same quadrant, as the conjunctiva was already surgically opened. After surgery, IOP remained under control for 5 months.

At the age of 18 months, IOP increased again to a level in the high 30s, despite the use of medication (travoprost and dorzolamide-timolol in fixed combination twice daily). A further EUA showed an encapsulated Tenon cyst, with correct position of the implant. As Tenon revision surgeries regularly need to be performed in adults, we decided to perform a second XEN revision surgery. However, while removing the scarring Tenon's layer, the implant was dislocated completely into the anterior chamber. Six months after implantation, the stent was still highly mobile inside the child's sclera. A new XEN gel stent (the third XEN gel stent) was implanted ab interno, as filtration and IOP control had been achieved in the previous months.

**Figure 2:**
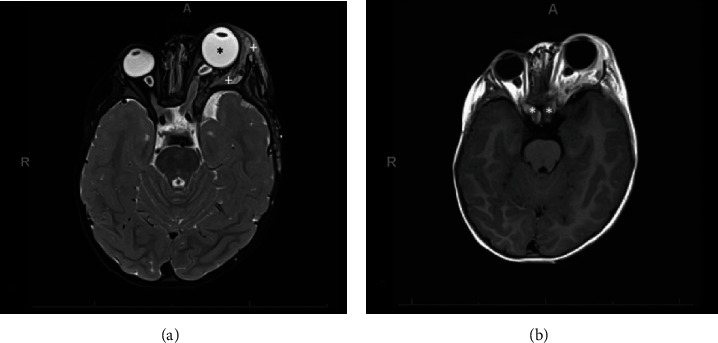
Magnetic resonance imaging. (a) Axial T2-weighted magnetic resonance image shows buphthalmos and exophthalmos of the left eye (asterisks denote the left eye). Also seen are neurofibromas in the left orbit, eyelid, and temple (plus signs denote the tumour). (b) Axial T1-weighted magnetic resonance image shows glioma of both optic nerves with diffuse enlargement (asterisks denote the tumours).

At that time, a progressive eyelid swelling became noticeable. A histopathological biopsy of the eyelid was taken, which revealed a spindle cell tumour and 5% KI 67 fraction positivity, indicating a perineural tumour. A further paediatric examination led to the suspected diagnosis of neurofibromatosis. A magnetic resonance imaging (MRI) scan revealed a retroocular tumour (suspected plexiform neurofibroma) OS with extraorbital portions in the eyelid and left temple (Figure 2). In addition, optic nerve gliomas on both optic nerves were found (Figure 2). The diagnosis of neurofibromatosis type 1 (NF1) was confirmed. Chemotherapy with carboplatin and vincristine along with mitogen-activated protein kinase (MEK) inhibitor therapy (trametinib) was initiated. MRI examinations and visual function tests were performed at 3-month intervals. Eye status and IOP were measured at least on a monthly basis.

IOP was under control for another 6 months with medication use but was then found again to be elevated to 32 mmHg. To reduce the burden of another surgical approach (e.g., shunt surgery) and because of the limited visual prognosis (only light projection), a laser ciliodestructive procedure was performed. Over the next 2 years, IOP ranged from 17 to 28 mmHg OS. Loss of light perception was documented during chemotherapy. Through until the last follow-up (at age 3 years), IOP was 28 mmHg OS under medication (travoprost and dorzolamide-timolol in fixed combination twice daily). No IOP-derived pain perception was apparent. A functional filtering bleb could not be seen.

## 3. Discussion

The management of congenital glaucoma is complex. Surgical intervention is considered the treatment of choice. Angle surgeries, including goniotomy and trabeculotomy (including 360° trabeculotomy using a microcatheter), are considered first-line procedures and may reach success rates of about 80% [1, 2, 6]. Filtering surgeries, such as trabeculectomy and glaucoma shunt surgery (i.e., Ahmed valve or Baerveldt implant), have been performed in the past and follow first-line treatment [3, 4, 7]. In children, success rates of trabeculectomy with MMC may reach 60% in the first years after surgery, but long-term complications, such as blebitis and endophthalmitis, may occur [3, 4]. Glaucoma drainage devices in paediatric patients can show a success ratio of about 50% after 10 years, with sight-threatening complications in 10% of cases [7].

The XEN gel stent may be an alternative surgical approach to other filtering procedures in challenging cases of congenital glaucoma, avoiding early complications due to overfiltration and with limited surgical trauma to the conjunctiva when using the ab interno approach. A case series of three children with congenital glaucoma has recently reported good outcomes of the XEN gel stent in 2 years of follow-up [8]. Case 1 showed successful IOP values after 2 years. In case 2, a combination of XEN gel stent and Kahook Dual Blade goniotomy was performed; a revision surgery with implantation of a second XEN gel stent was necessary because the stent was located within Tenon's layer. This new stent was implanted using a transconjunctival ab externo approach. Case 3 was a 10-year-old girl with complex secondary glaucoma and retinopathy of prematurity. During XEN stent implantation, a cataract surgery with iridectomy was performed simultaneously. IOP was well controlled, without any revision surgery after 2 years. Thus, no standard XEN implantation was performed in any of the cases.

In contrast, we failed to achieve long-term IOP control in our young patient after XEN gel stent implantation. In our perception, there is one major lesson to learn from our case. The elasticity and thickness of a child's Tenon's layer result in more difficult placement of the XEN gel implant. The implant may be repelled, even months after surgery, into the anterior chamber. Placement of the XEN implant above Tenon's layer, just under the conjunctiva, or a surgical incision of the conjunctiva with excision of Tenon's layer might be necessary for improved results.

No postoperative complications, such as cataract, ocular hypotony, choroidal detachment, or expulsive bleeding, were seen in our case. A larger case series would be necessary to evaluate the risks and benefits of the XEN gel implantation as a stand-alone procedure in congenital glaucoma in comparison with other filtering surgical procedures, such as trabeculectomy, after failed angle surgery. The main burden of filtering surgery in children (i.e., the high risk of conjunctival scarring) also remains a significant issue in XEN gel stent surgery.

The treatment of congenital glaucoma is even more complex when associated with systemic diseases such as NF1 [9–12]. Neurofibromatosis is a rare phakomatosis that mainly affects the skin and peripheral nervous system. There are two types: neurofibromatosis type 1 (NF1 or von Recklinghausen syndrome) and type 2, which is characterised by acoustic and/or central neurofibromatosis. Around 85% of patients suffer from NF1, as did our patient. Typical features of the disease are multiple neural tumours (neurofibromas), pigmented skin lesions (cafe au lait spots), and pigmented iris hamartomas (Lisch nodules). In addition, approximately 15%–20% of children with NF1 develop an optic pathway tumour, as did our patient [11, 12]. In our patient, increasing swelling of the eyelid began approximately 12 months after birth and was first considered to be secondary because of the initial exophthalmos. Eyelid biopsy at the age of 18 months and later MRI examination revealed the diagnosis NF1. Paediatric full body examination did not identify any pathology until that date. Unfortunately, vision-threatening optic nerve gliomas were found on both sides, posing a high risk of blindness.

To summarise, XEN gel stent surgery might be an alternative approach to conventional filtering techniques in congenital glaucoma after failed angle surgery. However, XEN placement and revision surgeries may be more challenging in infants than in adults. Further studies are needed to establish whether the XEN gel stent might offer any advantages over filtration surgeries. Until then, trabeculectomy should be considered a standard procedure after failed trabeculotomy. Moreover, little is known about the long-term survival of the XEN gel stent itself [13].
